# Preterm birth prevention—Time to PROGRESS beyond progesterone

**DOI:** 10.1371/journal.pmed.1002391

**Published:** 2017-09-26

**Authors:** Jane E. Norman, Phillip Bennett

**Affiliations:** 1 MRC Centre for Reproductive Health, University of Edinburgh, Edinburgh, United Kingdom; 2 Institute for Reproductive and Developmental Biology, Imperial College London, London, United Kingdom

## Abstract

In a Perspective, Jane Norman and Phillip Bennett argue that it is time to explore alternatives to progesterone for preventing preterm birth.

In this week’s *PLOS Medicine*, Crowther and colleagues publish the results of the PROGRESS Study, which examined the effect of maternal vaginal progesterone on the risk of respiratory distress syndrome in the baby [1]. Women at high risk of preterm birth because of a previous spontaneous preterm birth were recruited to this study, which involved self-administering 100 mg progesterone or a placebo vaginally from 20 to 34 weeks of pregnancy. No differences were found in the rate of the primary outcome (incidence of respiratory distress syndrome and severity of respiratory disease) between the progesterone and the placebo groups (incidence of respiratory distress syndrome in 42/402 [10.5%] and 41/388 [10.6%], respectively [RR 0.98, 95% CI 0.64–1.49]), nor were there differences in any of the other secondary outcomes. The study, a double-masked randomised placebo-controlled trial, was well conducted. About 40% of women who were approached agreed to participate. The primary outcome was available for all participating women, and around 65% of women were still taking study treatment by 34 weeks gestation. Rates of preterm birth were 36% and 17% before 37 and 34 weeks, respectively; hence, the recruitment strategy effectively identified a group at high risk. Although the study was terminated earlier than planned (after recruitment of 787 women rather than the 984 initially intended), and the incidence of the primary outcome was lower than anticipated in the placebo group (10.6% rather than 15%), it is unlikely that these issues have materially affected the conclusions of the study.

Although vaginal progesterone is not licensed for prevention of preterm birth, it has been widely adopted by clinicians around the world for this purpose. Progesterone for preterm birth prevention in high-risk women is endorsed by expert guideline groups [2], including the National Institute for Clinical Excellence (UK) [3] and the FIGO Working Group on Best Practice in Maternal-Fetal Medicine [4]. The PROGRESS Study is the third large randomized trial (the others being our own OPPTIMUM study [5] and the study of O’Brien et al. [6]) to show a lack of efficacy of vaginal progesterone. Specifically, the PROGRESS trial showed no difference between the 2 groups in gestation of delivery, preterm birth before 37 weeks, or preterm birth before 34 weeks. We believe it is now time for a comprehensive review of the data on progesterone to identify risks and benefits in women at risk of preterm birth for different reasons.

Ostensibly, there is biological plausibility behind the administration of progesterone for prevention of preterm birth. Progesterone can inhibit contractions of the myometrium in vitro [7], and antiprogestogens are used as abortifacients. However, progesterone levels are high during pregnancy, far above receptor saturation. There is no evidence that women delivering preterm have lower progesterone levels and administration of progesterone vaginally does not increase circulating concentrations. Therefore, the mechanism by which a modest additional amount of progesterone could achieve a therapeutic effect is unclear.

In evaluating progesterone to prevent preterm birth, the Cochrane analysis groups evidence by maternal risk factor for preterm birth rather than by route of administration or type of progesterone [8], even though vaginal natural progesterone and intramuscular 15 dihydroprogesterone caproate are very different therapies. The Cochrane analysis suggests that, in women with a previous preterm birth, progestogens reduce perinatal mortality and rates of preterm birth before 34 weeks, low birthweight, and neonatal death. Inclusion of women from OPPTIMUM and PROGRESS will double the existing sample size; a major impact on the treatment effect and confidence intervals is to be expected. In women at risk of preterm birth because of a short cervix, Cochrane suggests that progesterone reduces rates of preterm birth before 34 weeks but has no impact on perinatal mortality, low birthweight, or neonatal death [8]. A single randomized study and an individual patient data meta-analysis have shown that progesterone in women with a short cervix reduces a composite adverse neonatal outcome [9,10], although the individual study was insufficient to persuade the United States Food and Drug Administration (FDA) to license vaginal progesterone. Importantly, the FDA considered that a more robust statistical approach failed to result in a statistically significant effect of progesterone [11]. To address this complexity, the Patient-Centered Outcomes Research Initiative (PCORI) has initiated an individual patient data meta-analysis that will be conducted by an independent data centre. It is hoped that this will address the uncertainty about the efficacy of progesterone to prevent preterm birth in women with various risk factors.

Even if progesterone is found to reduce preterm birth rates in women with either a previous preterm birth or a short cervix, it is increasingly evident that alternatives are needed because the impact on preterm birth rates overall are small. Modelling of the impact of progesterone to prevent preterm birth in women with a previous preterm birth (but without a short cervix), and assuming a 20% reduction in preterm birth before 37 weeks, suggests an absolute reduction of 0.01% in preterm birth rates [12]. It has been claimed that routine cervical length screening of the entire US pregnant population, and offering vaginal progesterone to all women with a cervical length of 10–20 mm, would be cost effective [13]. However, in this scenario preterm birth would be prevented in only 913 women. This represents an absolute risk reduction of 0.02%, given that in 2015 there were 382,786 preterm births in the US [14].

There has been huge renewed excitement amongst clinicians and the pharmaceutical industry for the use of progestogens to prevent preterm birth in the 21st century. A PubMed search shows 792 publications on this topic since 2000. The PROGRESS Study adds to a body of evidence suggesting that the role of progesterone may be more limited than previously thought. Preterm birth remains the single biggest cause of neonatal mortality and morbidity worldwide, and effective therapeutic interventions are desperately needed. It is time to start exploring alternatives, and to progress beyond progesterone for the prevention of preterm birth.

## Abbreviation

FDA: United States Food and Drug Administration
